# At-home HIV self-testing during COVID: implementing the GetaKit project in Ottawa

**DOI:** 10.17269/s41997-021-00505-8

**Published:** 2021-05-17

**Authors:** Patrick O’Byrne, Alexandra Musten, Lauren Orser, Gauri Inamdar, Marie-Odile Grayson, Clay Jones, Megan Francoeur, Sarah Lachance, Vickie Paulin

**Affiliations:** 1 School of Nursing, University of Ottawa, 451 Smyth Road, Ottawa, Ontario K1H 8M5 Canada; 2 Infectious Diseases and Sexual Health Services, Ottawa Public Health, Ottawa, Canada; 3 Ontario HIV Treatment Network, Toronto, Canada

**Keywords:** Access, HIV, Self-testing, Implementation, Accès, VIH, autodépistage, implémentation

## Abstract

**Setting:**

In March 2020, COVID-19 shuttered access to many healthcare settings offering HIV testing and there is no licensed HIV self-test in Canada.

**Intervention:**

A team of nurses at the University of Ottawa and Ottawa Public Health and staff from the Ontario HIV Treatment Network (OHTN) obtained Health Canada’s Special Access approval on April 23, 2020 to distribute bioLytical’s INSTI HIV self-test in Ottawa; we received REB approval on May 15, 2020. As of July 20, 2020, eligible participants (≥18 years old, HIV-negative, not on PrEP, not in an HIV vaccine trial, living in Ottawa, no bleeding disorders) could register via www.GetaKit.ca to order kits.

**Outcomes:**

In the first 6 weeks, 637 persons completed our eligibility screener; 43.3% (*n* = 276) were eligible. Of eligible participants, 203 completed a baseline survey and 182 ordered a test. These 203 participants were an average of 31 years old, 72.3% were white, 60.4% were cis-male, and 55% self-identified as gay. Seventy-one percent (*n* = 144) belonged to a priority group for HIV testing. We have results for 70.9% (*n* = 129/182) of participants who ordered a kit: none were positive, 104 were negative, 22 were invalid, and 2 “preferred not to say”; 1 participant reported an unreadiness to test.

**Implications:**

Our results show that HIV self-testing is a pandemic-friendly strategy to help ensure access to sexual health services among persons who are good candidates for HIV testing. It is unsurprising that no one tested positive for HIV thus far, given the 0.08% positivity rate for HIV testing in Ottawa. As such, we advocate for scale-up of HIV self-testing in Canada.

## Résumé

**Contexte:**

En mars 2020, la COVID-19 a empêché l’accès à de nombreux établissements de santé offrant des tests de dépistage du VIH et il n’existe pas d’autotest du VIH autorisé au Canada.

**Intervention:**

Une équipe d’infirmières de l’Université d’Ottawa et de Santé publique Ottawa et le personnel du Réseau ontarien de traitement du VIH (OHTN) ont obtenu l’approbation d’accès spécial de Santé Canada le 23 avril 2020 pour distribuer l’autotest VIH INSTI de bioLytical à Ottawa; nous avons reçu l’approbation du Comité d’éthique de la recherche (CER) le 15 mai 2020. À partir du 20 juillet 2020, les participants admissibles (≥18 ans, séronégatifs, ne prenant pas de PPrE, ne participant pas à un essai de vaccin contre le VIH, vivant à Ottawa, ne souffrant pas de troubles de la coagulation) pouvaient s’inscrire via www.GetaKit.ca pour commander une trousse d’autotest.

**Résultats:**

Au cours des six premières semaines, 637 personnes ont rempli notre questionnaire d’admissibilité; 43,3 % (*n* = 276) étaient admissibles. Parmi les participants admissibles, 203 ont répondu à l’enquête de référence et 182 ont commandé un test. Ces 203 participants avaient en moyenne 31 ans, 72,3 % étaient blancs, 60,4 % étaient hommes-cis et 55 % s’identifiaient comme gays. Soixante et onze pour cent (*n* = 144) appartenaient à un groupe prioritaire pour le dépistage du VIH. Nous avons les résultats pour 70,9 % (*n* = 129/182) des participants qui ont commandé une trousse : aucun n’était positif, 104 étaient négatifs; 22 étaient invalides; 2 « préféraient ne pas le dire »; et un seul a indiqué qu’il n’était pas prêt à subir le test.

**Conclusion:**

Nos résultats montrent que l’autodépistage du VIH est une stratégie adaptée à la pandémie pour aider à garantir l’accès aux services de santé sexuelle aux gens qui sont de bons candidats au dépistage du VIH. Il n’est pas surprenant que personne n’ait été testé positif pour le VIH jusqu’à présent, étant donné le taux de positivité de 0,08 % pour le test de dépistage du VIH à Ottawa. C’est ce qui nous incite à préconiser l’intensification du recours à l’autotest du VIH au Canada.

## Introduction

Following the World Health Organization’s March 11, 2020 declaration that COVID was a pandemic, the Ontario Ministry of Health and Long-Term Care (2020) restricted most non-essential healthcare on March 17, 2020. This resulted in near-immediate closure of most HIV testing locations or major restrictions on the few sites that remained open (CAS, 2020). Despite such emergency orders, people continued to engage in sexual contact with new partners, creating a situation of potential ongoing HIV transmission and a lack of testing (Shilo & Mor, 2020). Due to the harms associated with delayed HIV diagnosis and the fact that public health messaging has long rejected a reliance on symptoms to guide testing (CDC, 2017; May, 2017; O’Byrne & Orser, 2020), a group of nurses from the University of Ottawa (School of Nursing) and Ottawa Public Health, plus staff from the OHTN, established the first free HIV self-testing program in Canada.

Our objective was to increase testing for the approximately 14% of Canadians estimated to not know they are HIV-positive (PHAC, 2018), while maintaining physical distancing and limiting healthcare system burden during the COVID pandemic. Specifically, we sought to maintain testing because diagnosis is the foundation of the UNAIDS 90-90-90 strategy, in which 90% of persons living with HIV are diagnosed, 90% of those diagnosed are linked to care, and 90% of those linked to care achieve viral suppression. As part of this, we targeted persons most affected by HIV (hereafter “priority groups”), including gay, bisexual, and other men who have sex with men (gbMSM); transgender persons; persons of African, Black, or Caribbean (ACB) ethnicities; members of Indigenous communities; persons who use injection drugs (IDU); racialized minorities; and persons from regions where HIV is endemic (OHESI, 2019; Friedman et al., 2017). Our goals were (1) to make HIV testing available to priority groups, without limiting tests to these groups, and (2) to evaluate how many members of these groups would use self-testing. That is, would unrestricted HIV self-testing be used by members of HIV priority groups, at what rates, and with what outcomes? In this paper, we overview our HIV self-testing project and present the first 6 weeks of data.

## Methods

When we implemented GetaKit, HIV self-testing was not licensed in Canada. We thus obtained approval on April 23, 2020 from Health Canada through its special access program to distribute bioLytical INSTI® HIV Self-Tests in Ottawa. We purchased these kits for $20 each. We then developed pre-/post-test counselling materials and a website for ordering (www.GetaKit.ca), which included resources and information about HIV and instructions (including videos) about self-testing. This was undertaken in partnership with the AIDS Committee of Ottawa and MAX Ottawa. We also established linkage-to-care pathways, working with local primary care offices, HIV testing clinics, infectious disease physicians, and emergency departments. The University of Ottawa REB approved this study on May 15, 2020. Implementation began on July 20, 2020.

### The HIV Self-Test

The INSTI® HIV Self-Test is intended for lay persons and functions as “a single use, rapid, flow-through in vitro qualitative immunoassay for the detection of antibodies to HIV Type 1 (HIV-1) and Type 2 (HIV-2) in human fingerstick whole blood” (WHO, 2018). This self-test is a modification of its parent product, the INSTI® HIV Antibody Test for professional use, which is Health Canada approved. The test principle and mechanism are identical for both. The INSTI® HIV Self-Test is CE (“Conformitè Europëenne”) marked, prequalified by the WHO, and approved for use in Kenya, Nigeria, and Vietnam (IAS, 2019; UNAIDS, 2017; Unitaid, 2018; WHO, 2018).

Like the INSTI® HIV Antibody Test, the INSTI® HIV Self-Test has a quality assurance mechanism, whereby a “control” dot is visible when the test is performed correctly. The test result is invalid if this dot is not present. This shows people who self-test when the result is valid.

The performance and usability of INSTI® Self-Test has been evaluated in studies with lay users in South Africa (HSTAR I and II), Kenya (KEMRI), and Congo Brazzaville (Bwana et al., 2018). The study results from the South Africa (HSTAR II) study found that 99.4% of participants said the device was easy to use, 99.7% successfully completed testing, and 97.3% would recommend it to others. Comparison of the performance efficacy analysis of INSTI® HIV Self-Test from the KEMRI, HSTAR III, and Congo Brazzaville studies is provided in Table 1.

**Table 1:** Sensitivity and specificity of the INSTI® HIV Self-Test

	KEMRI	HSTAR003	Congo Brazzaville
Sensitivity	98.51%	98.98%	100%
Specificity	99.26%	100%	100%

### The GetaKit package

**Fig. 1:**
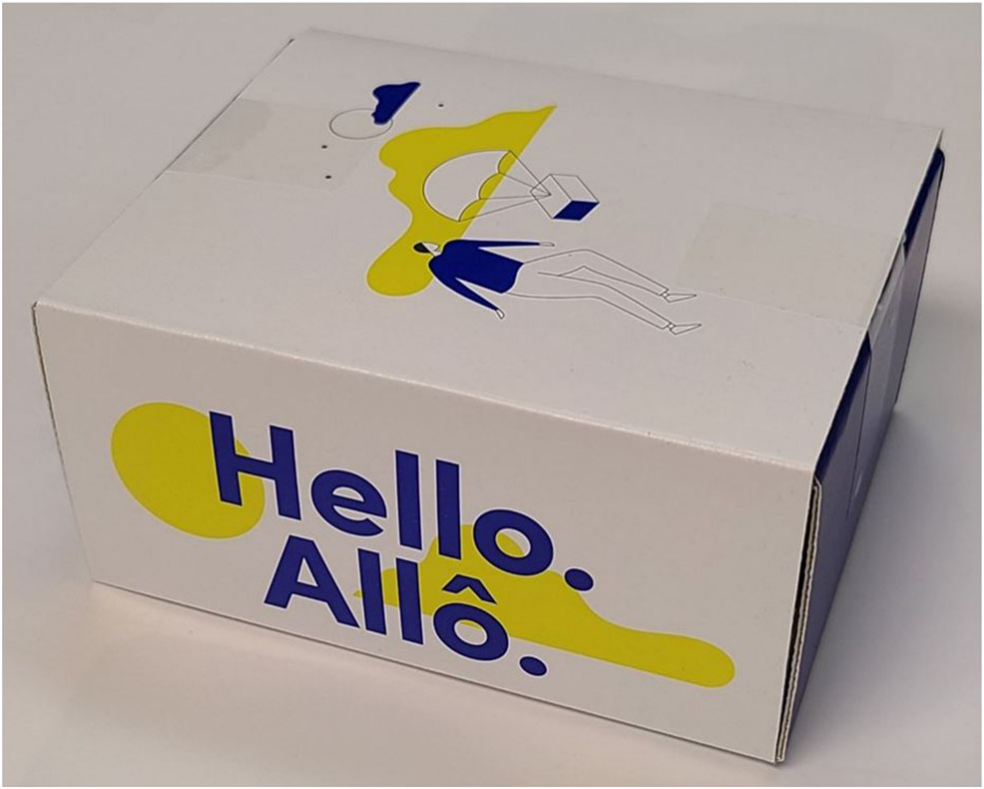
GetaKit exterior

**Fig. 2:**
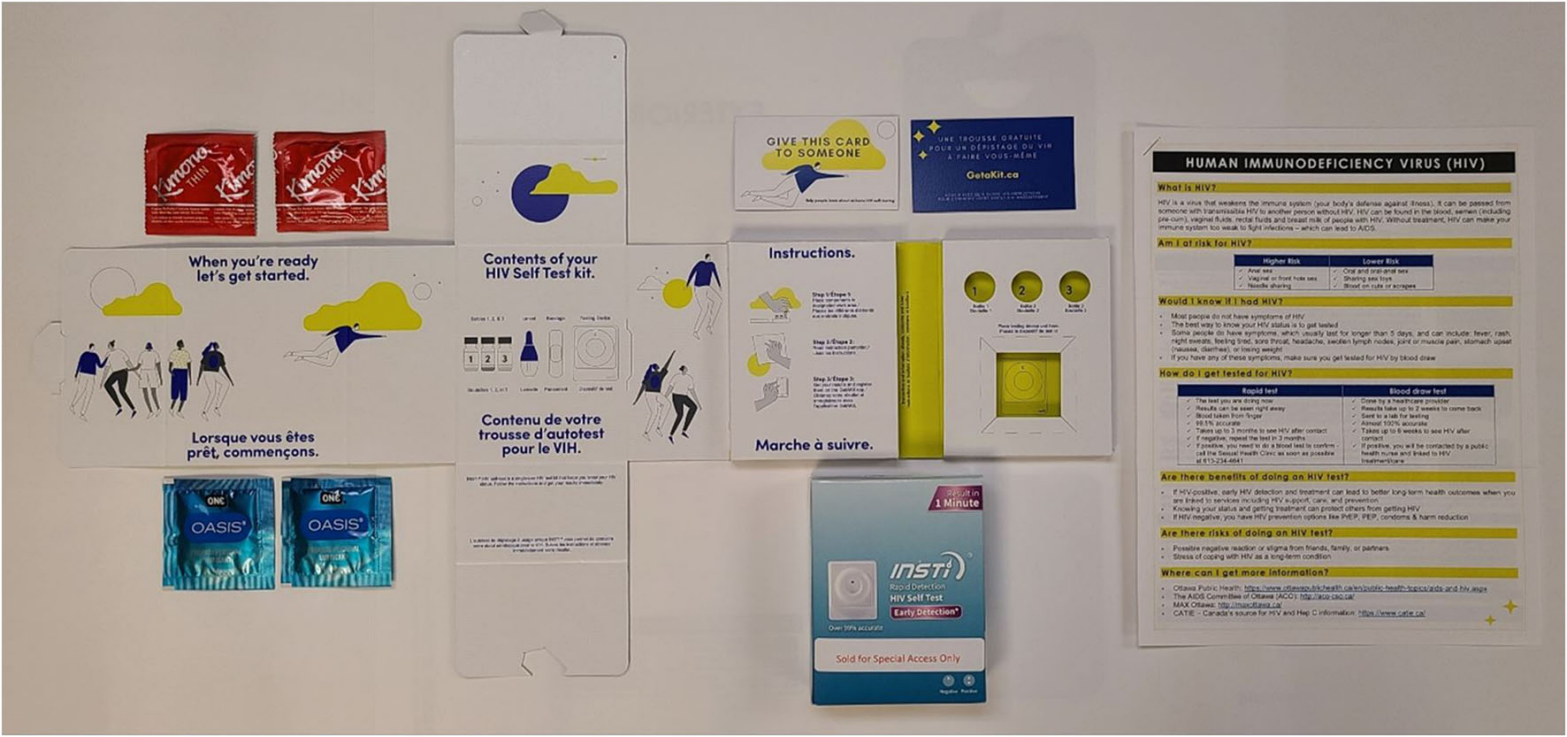
GetaKit interior with contents

For GetaKit, we developed a shipping box to distribute the self-test. While this kit had no information on its exterior, inside we provided information and forms addressing pre-/post-test counselling, self-testing, and support services. We also included lubricated condoms, packages of lubricant, and promotional cards to give to others to inform them about self-testing. All materials in and on the GetaKit box were in French and English. Figures 1 and 2 show the kit and its contents.

### Recruitment

We disseminated information about GetaKit in Ottawa via social media (Instagram, Twitter, Facebook), traditional media (Ottawa Citizen, CBC, Ottawa Sun), through posters (in the local STI/HIV testing clinic, sex clubs, gay bars, and poster collars on downtown streets and in the Gay village), and through community organizations (AIDS Committee of Ottawa and MAX Ottawa). We also informed primary care providers. Last, we targeted community groups, healthcare clinics, and physical locations that were frequented by HIV priority groups in Canada.

### Eligibility

Eligible persons needed to be HIV-negative, ≥18 years of age, live in Ottawa, and have a personal email address and cellular phone number. Exclusion criteria included using HIV pre-exposure prophylaxis (PrEP), being in an HIV vaccine trial, and having a bleeding disorder.

### Self-test pathway

**Fig. 3:**
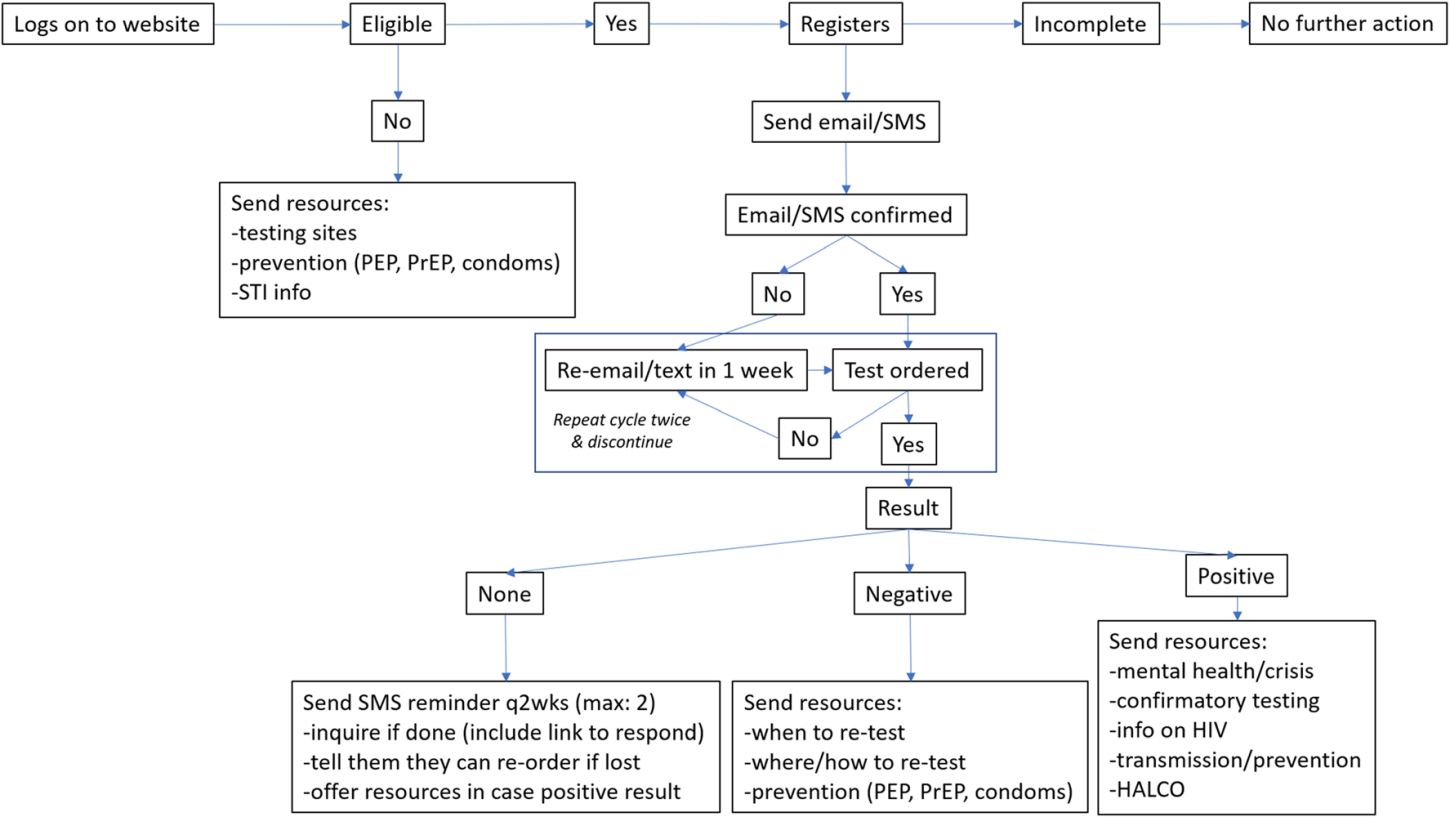
GetaKit pathway

Anyone interested in self-testing could visit www.GetaKit.ca and complete a series of steps (Fig. 3). The first was the eligibility screener. Those deemed ineligible were diverted to resources on the website for in-person testing, counselling, and support. Those who were eligible were asked to register and sign a consent form to use the self-testing device as part of research. For registration, participants had to input an authentication code that was sent to their cellular phone. Once confirmed, the person could complete a baseline survey (for which they were compensated with a $10 coffee gift card) and order a kit, which arrived in 2–3 business days by mail. The delivery cost was $5–10 per kit, depending on courier and location.

If the participant did not report a result after 1 week from ordering, we sent a reminder to do so. If the participant reported a negative result, we informed him/her/them about retesting based on window periods, to seek care if symptoms develop, and about post-exposure prophylaxis. We also offered an appointment in our nurse-led PrEP clinic (PrEP-RN) for anyone who reported a negative test result and was gbMSM, ACB, IDU, transgender, from an Indigenous community, a racialized minority, or from an HIV-endemic region. PrEP linkage to care and follow-up occurred via established protocols (O’Byrne et al., 2019). In the event of a positive result, our path was to immediately reach out by telephone to assess the person, to provide counselling and support, and to schedule confirmatory testing. We would refer anyone with positive serology for care.

### Data collection

We collected data from the eligibility and baseline surveys, the kit order form, and results reporting page. While all components of the eligibility survey were mandatory, people could opt out of most questions in the baseline survey and could choose “prefer not to answer” when submitting their test results.

### Data analysis

Data analysis involved extracting participants’ responses from the website into Excel files. Once obtained, we analyzed the database using descriptive statistics. Our objectives were to determine initial uptake, including total number and description of participants, as well as test results (number and outcome). One key aspect of our analysis was to determine the proportion of participants who belonged to HIV priority groups in Canada.

## Results

Our results have three sections: (1) project uptake, (2) participant characteristics from the baseline survey, and (3) test results.

### Project uptake

Between July 20 and August 30, 2020, 637 persons completed the eligibility screener; 43.3% (*n* = 276/637) were eligible. The most common reasons for ineligibility were not living in Ottawa (*n* = 122), taking PrEP (*n* = 64), not giving a phone number (*n* = 35), and being <18 years old (*n* = 21). Among eligible participants, 73.6% (*n* = 203/276) completed a survey and 90% (*n* = 182/203) ordered a test. From those who ordered tests, 70.9% (*n* = 129/182) reported results. One participant selected “prefer not to report” for all survey questions and was excluded from analysis.

### Participant characteristics

The 202 participants who completed our baseline survey were on average 31 years old. They were also 72.3% (*n* = 146/202) white, 60.4% (*n* = 122/202) cis-male, 2% (*n* = 4/202) transgender, 55% (*n* = 111/202) gbMSM, 78.7% (*n* = 159/202) Canadian born, and 3% (*n* = 6/202) Indigenous (First Nations, Inuit, Métis). When we analyzed to see how many participants had at least one characteristic of an HIV priority group, 71.3% (*n* = 144/202) did (i.e., were gbMSM, ACB, a racialized minority, IDU, transgender, Indigenous, and/or born where HIV is endemic). In contrast, when we analyzed for the cluster of characteristics that made participant the lowest risk for HIV, we identified that only 18.8% (*n* = 38/202) of participants were white, cis-gender, heterosexual, and born in Canada (8 were cis-male, 30 were cis-female). For income (CAD), 15.8% (*n* = 32/202) reported <$20,000, 12.9% (*n* = 26/202) reported $20,000–$40,000, 30.2% (*n* = 61/202) reported $40,000–$75,000, and 31.2% (*n* = 63/202) reported income >$75,000; 9.9% (*n* = 20/202) did not answer. See Table 2.

**Table 2:** Participants

Item	Subcategory	%	Number (*n* = 202)
Ethnicity	White	72.3%	146
	Black	3.5%	7
	Southeast Asian	7.4%	15
	South Asian	3.5%	7
	Indigenous	3%	6
Gender	Cis-male	60.4%	122
	Cis-female	35.6%	72
	Transgender	2%	4
Sexual orientation	gbMSM	55%	111
	Lesbian and WSW	15.3%	31
	Heterosexual (male)	7.4%	15
	Heterosexual (female)	20.3%	41
Income (CAD)	<$20,000	15.8%	32
	$20,000–$40,000	12.9%	26
	$40,000–$75,000	30.2%	61
	>$75,000	31.2%	63

*gbMSM*, gay, bisexual, and other men who have sex with men; *WSW*, women who have sex with women

Only 35.6% (*n* = 72/202) of participants had heard of HIV self-testing. Of those unaware of HIV self-testing (*n* = 128), 64% (*n* = 82/128) belonged to HIV priority groups. For testing history, 27.2% (*n* = 55/202) reported no prior HIV screening. Among participants never previously tested for HIV, 56.4% (*n* = 31/55) belonged to HIV priority groups. Among those who had never tested before (*n* = 55), the non-exclusive reasons for no prior testing were as follows: 69% (*n* = 38/55) felt they were at low risk (with 20 of these participants identifying as heterosexual, 9 as gbMSM, and 6 as lesbian); 16.4% (*n* = 9/55) were afraid to find out their HIV status; 7.3% (*n* = 4/55) did not have time; and 20% (*n* = 11/55) did not know where to access testing. Among those who had previously undergone HIV testing (*n* = 147), 38.8% (*n* = 57/147) had not been tested within the previous 12 months. Regarding reasons for testing, 40.6% (*n* = 82/202) wanted testing due to condomless sex, 5% (*n* = 10/202) reported that a condom had broken during sex, and 33.7% (*n* = 68/202) provided qualitative answers (focusing around knowledge about HIV status, peace of mind, and risk practices by themselves/partners); an additional 19.8% (*n* = 40/202) “preferred not to answer”.

Excluding marijuana (which 74.3%, *n* = 150/202, of the sample reported), 46.5% (*n* = 75/202) reported a history of recreational drug use, with cocaine being most common: 24.3% (*n* = 49/202) of our sample reported cocaine use. Additionally, 7.9% (*n* = 16/202) reported narcotic use including fentanyl, and 5.9% (*n* = 12/202) reported crystal meth use, with 66.7% (*n* = 8/12) of those reporting meth use being gbMSM.

### Test results

Overall, 128 participants reported test results for a response rate of 70.9% (*n* = 129/182). However, because 1 participant reported he was not ready to perform the test and 1 kit was undeliverable, the adjusted response rate is 71.7% (*n* = 129/180). Of these results, none were positive, 0.8% (*n* = 1/129) was “I’m not ready to test yet”, 1.6% (*n* = 2/129) were “prefer not to report”, 82.2% (*n* = 106/129) were negative, and 17.1% (*n* = 22/129) were invalid. Communication with 14 participants who reported invalid results revealed their belief they had not provided an adequate fingerstick blood sample. We sent new tests to these participants; 10 reported subsequent negative results. Notably, our number of invalid results may be higher than established in the published literature because we required that participants report invalid results before obtaining a second kit; in contrast, we did not require participants to report negative results until they ordered another test kit, which could not occur more frequently than every 3 months. As such, we may have a more accurate representation of invalid results, but underreporting of negative results, although even if all unreported results were negative, our invalid rate would still be approximately 11% (*n* = 22/180). Last, among these 128 participants, 63.6% (*n* = 82/129) belonged to an HIV priority group and were offered a referral for HIV PrEP.

## Discussion

Herein, we reported on our GetaKit project, which launched during COVID as the first free at-home HIV self-testing project in Canada. During the first 6 weeks of implementation, about half of the participants who were eligible for an HIV self-test ordered one and about half who ordered a kit reported results, none of which were positive. We also found that nearly three quarters of our sample belonged to HIV priority groups. Last, we identified that two thirds of those who reported results were eligible for PrEP—which we offered. These results raise a few points for discussion.

One noteworthy point is that our findings align with the literature about the uptake and use of HIV self-testing. That 637 persons sought to determine their eligibility, 203 registered, and 182 (90% of those eligible) ordered kits suggests that our self-testing program was acceptable. Reinforcing this assertion is that we observed this rate of interest and uptake within the 6 weeks of project implementation, despite only one third of our sample having previously heard of HIV self-testing. Such findings about acceptability have emerged in other studies, which have also shown self-testing to be preferred over facility-based testing (Chen et al., 2010; Lee et al., 2013; Bilardi et al., 2013; Medline et al., 2017; Hurt et al., 2016; Rosales-Statkus et al., 2015). Explanations in the literature about preferences for self-testing are that it increases autonomy by allowing people to test when, where, and with whom they choose (Qin et al., 2018). The literature also suggests that participants find self-testing less stigmatizing (Johnson et al., 2020; Qin et al., 2018). Considering that HIV priority groups often face high rates of discrimination within healthcare (Zeeman et al., 2018), it is perhaps unsurprising that uptake among these groups was elevated, as self-testing creates a way to screen for HIV without potential scrutinization by healthcare providers. Conversely, more testing among these persons may relate to the fact that we specifically targeted these groups.

As well, our results align with the literature on HIV self-testing among first-time testers, with rates of first-time testers ranging up to one third of participants (CDC, 2019; Dean et al., 2019; Clark et al., 2019; Johnson et al., 2020; Greacen et al., 2013). In our study, one quarter of our sample had never done HIV testing before, with over half belonging to HIV priority groups.

Where our results diverge from the literature is that despite our sample including many first-time testers from priority groups, our positivity rate was 0% from the 129 of 180 potentially reportable results. This finding, however, may not be surprising, as the positivity rate for HIV testing in 2018 (last year of available data) was 0.08% for Ottawa and 0.10% for Ontario, which would equate to 1 positive result per 1250 tests in Ottawa and 1 positive result per 1000 tests in Ontario. As such, we cannot compare positivity results until we exceed 1250 tests. Compounding this, however, was our response rate for results, at about 70%, which matches those found in the literature (MacGowan et al., 2019; Ricca et al., 2016). This highlights that we may need to perform more tests to identify positive results within our sample, as nearly one third of test results were not reported. While this reporting rate is not inherently a problem, it may skew cost-effectiveness and public health analyses, which focus on positivity rates as an important metric. That only 70% of participants reported their results also highlights the need to engage in data collection about strategies that could be developed and implemented to increase the rate of reported results. Such initiatives should occur, however, with the awareness that a reluctance to report results may, in fact, be a beneficial strategy to increase the uptake of HIV testing more broadly. As a final point, it is also possible that a participant may have received a positive result among those who either “preferred not to report” their result or among those who did not report their results.

Last, one striking finding was the 17.1% of reported test results that were invalid. While previous studies have found that over 95% of participants could successfully engage in HIV self-testing (Bwana et al., 2018; Figueroa et al., 2018), we quickly established that changes needed to be made to the instructions about how to perform self-testing to obtain a satisfactory blood sample and a valid result. At a cost of $20 per test and $5–10 shipping per kit, the cost of invalid results was high (i.e., $25–30 per invalid result that was repeated), and efforts needed to be made to improve these outcomes and costs. This was an unexpected finding that required modification. Other changes that we identified would be to expand the validation process to include email, add community-based locations, such as local AIDS service organizations and pharmacies, where registration could occur (for those without cellular and Internet access), and add established pick-up locations (so participants do not have to use a personal address). In combination, these strategies might help further increase the uptake of HIV self-testing.

### Limitations

First, our data arose from self-report and participants’ abilities to engage in HIV self-testing. Testing during window periods and false negative results were possible. We need to observe the within-person results over time to determine whether time of testing would affect our results. Second, GetaKit operated in one city of one million persons, where there were established HIV testing clinics and a specialized gbMSM testing clinic (see O’Byrne et al., 2014). Uptake of self-testing could be higher in cities where access to testing is less available. Third, we implemented GetaKit during the COVID pandemic, when access to HIV testing was restricted overall. While our clinic continued to operate, services were reduced for persons who were accustomed to accessing specialized HIV testing clinics. As such, a lack of access to traditional testing services may have increased demand for self-testing, potentially inflating interest in and acceptability of this testing approach. Fourth, over half of our participants had an income greater than $41,000 per annum and all had cellular phone and Internet access. If our findings would be replicated with other groups who are at risk for HIV but of lower socio-economic status requires more research.

## Conclusion

Being the first at-home HIV self-testing program in Canada, GetaKit showed that self-testing is a viable solution to have persons who have never previously tested for HIV do so. GetaKit also showed that self-testing is one way to maintain access to HIV testing during pandemics. Important points that arose from our implementation data, however, were a 0% positivity rate, a 17.1% rate of invalid results, and that only two thirds of participants reported results. Future efforts should work to decrease invalid results to improve testing experiences for participants, to obtain accurate results, and to ensure effective use of resources. In contrast, while efforts to increase the rates at which participants report results could be a useful metric, it may not correspond with the ultimately desired outcome of self-testing; i.e., to have at least 90% of people living with HIV know they are HIV-positive. Stated otherwise, mandating that persons who self-test report their results might undermine people’s willingness to self-test, thus impeding the actual goal of HIV testing: to increase HIV status awareness, connect people to care, improve quality and quantity of life, and decrease onward transmission.

Taken as a whole, therefore, considering that approximately 14% of persons in Canada are unaware they are HIV-positive, despite ongoing free access to HIV testing in clinical and outreach settings, and because the basis of the UNAIDS 90-90-90 goal relies on people knowing they are HIV-positive, GetaKit adds more evidence that self-testing is an important strategy to add to existing HIV testing programs, both during COVID and afterward. Our ongoing delivery of GetaKit and future publications on this matter will add more valuable information to help better understand HIV self-testing in Canada.

### Implications for policy and practice

What does this study add to existing knowledge?

- This project is the first mail-out HIV self-testing program in Canada.
- This project created a novel access point for HIV testing during COVID when healthcare was mostly inaccessible.

What are the key implications for public health interventions, practice or policy?

- Further research is required to determine how to maximize uptake of HIV self-testing among the persons most affected by HIV.

## Data Availability

Not applicable.
